# Development and verification of a prognostic nomogram model for triple-negative breast cancer patients with neoadjuvant therapy

**DOI:** 10.3389/fonc.2026.1850253

**Published:** 2026-05-28

**Authors:** Yuehua Zhang, Xinzhong Zhang, Xinfeng Wang, Yuling Zhang, Xiance Tang, Zhenxin Zhang, Hongqiang Guo

**Affiliations:** 1 Department of Internal Medicine, The Affiliated Cancer Hospital of Zhengzhou University & Henan Cancer Hospital, Zhengzhou, China; 2 Follow-up Center, The Affiliated Cancer Hospital of Zhengzhou University & Henan Cancer Hospital, Zhengzhou, China; 3 Information Center, The Affiliated Cancer Hospital of Zhengzhou University & Henan Cancer Hospital, Zhengzhou, China

**Keywords:** neoadjuvant therapy, nomogram, predictive factor, prognosis, triple-negative breast cancer

## Abstract

**Background:**

A favorable prognostic nomogram model for predicting overall survival (OS) and stratifying prognostic risk in triple-negative breast cancer (TNBC) patients treated with neoadjuvant therapy (NAT) is lacking. The aim of this study was to formulate an excellent model for predicting the prognosis of these patients.

**Methods:**

From January 2018 to December 2024, 804 patients with TNBC who underwent NAT at Henan Cancer Hospital were included. Patients were randomly assigned to a training cohort (n = 603) or an internal validation cohort (n = 201). A prognostic nomogram model was developed and validated. Predictive performance and discrimination were further measured and compared with other models.

**Results:**

Age, pretreatment tumor size, pretreatment lymph node metastasis, pathological status, skin invasion, and lymphovascular invasion (LVI) were identified as independent prognostic factors and were included in the construction of a prognostic model for TNBC treated with NAT. The time-dependent receiver operating characteristic (ROC) and the C-index of our model consistently outperformed other prognostic factors in both the training and internal validation cohorts. Using the optimal cut-off values (90 and 160) selected by X-tile, patients were stratified into low-risk (total points ≤ 90), moderate-risk (90 < total points ≤160), and high-risk (total points > 160) groups with significantly different OS.

**Conclusions:**

In patients with TNBC, the large sample nomogram model, including age, pretreatment tumor size, pretreatment lymph node metastasis, pathological status, skin invasion, and LVI, was demonstrated to predict the survival of individuals with favorable performance and discrimination. Furthermore, risk stratification derived from the model can identify unfavorable survival in early-stage TNBC, guide personalized treatment strategies, and select comparable study cohorts in clinical trials.

## Introduction

Triple-negative breast cancer (TNBC) accounts for 10%-20% of all breast cancers (BCs) and is considered the most aggressive subtype of BC (1, 2). It is characterized by a lack of immunostaining for estrogen, progesterone, and human epidermal growth factor receptor (1, 3). Over the past decade, the treatment strategy for operable TNBC has shifted from surgery followed by adjuvant therapy to neoadjuvant therapy (NAT) before surgery, especially for patients with stage II-III disease (4–7). NAT can downstage the tumor stage, increase the breast conservation rate, and improve the prognosis. However, TNBC patients receiving NAT show great heterogeneity of disease with significantly different survival (8, 9), rendering it critical to develop a risk stratification model.

Currently, there are no widely approved biomarkers for predicting the prognosis of patients with TNBC and NAT. Therefore, identifying biomarkers that can distinguish between favorable and unfavorable responses is crucial for treatment decisions in patients with TNBC. Pathological complete response (pCR) is a widely used and effective method for evaluating the efficacy of NAT, which can significantly improve survival outcomes in patients with BC patients (7, 10). However, some studies indicate that increasing pCR rates do not necessarily correlate with improved prognosis, as a small proportion of patients who achieve pCR may still experience distant metastasis (11). Consequently, predicting the efficacy and prognosis of NAT requires more comprehensive analysis.

Moreover, individualized prediction is regarded as an important requirement for an excellent predictive model, considering that prognosis substantially affects the quality of life of patients with TNBC. More importantly, a prognostic model with excellent discrimination performance can help develop new drugs for high-risk populations. Therefore, it is crucial for clinicians to construct a novel model that accurately stratifies the prognostic risk.

In the present study, we analyzed data from 804 TNBC patients who received NAT to develop a prognostic nomogram model that can be applied to predict individual prognosis and separate patients into different risk groups to help physicians estimate overall survival (OS) in TNBC with NAT.

## Methods

### Study design and patients

In this study, we collected the clinical and pathological data of 804 patients diagnosed with TNBC who received NAT at Henan Cancer Hospital from 2018-2024. Data, including age, menopausal status, primary site, pretreatment tumor size, pretreatment lymph node metastasis, clinical TNM staging, pathological information, and treatment details, were retrospectively obtained from patient records. Clinical TNM staging was assessed using the eighth edition of the AJCC breast cancer staging system before NAT (12).

The inclusion criteria were as follows (1): histological confirmation of TNBC and (2) received NAT. The exclusion criteria were as follows: (1) bilateral TNBC, (2) metastatic TNBC, (3) not receiving mastectomy, and (4) incomplete follow-up.

**Figure 1:**
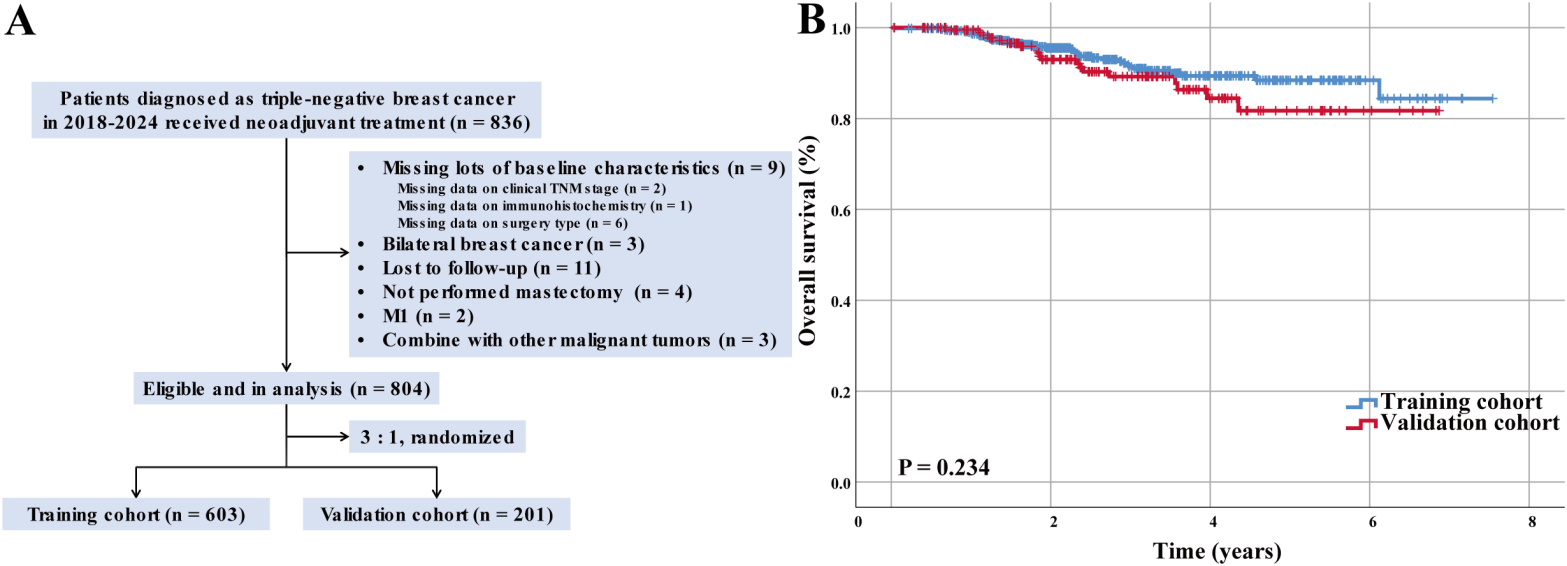
Baseline clinicopathologic features in the current study. **(A)** Flowchart of participant enrollment. **(B)** Kaplan-Meier survival analysis of training cohort and validation cohort.

Ultimately, 804 patients were enrolled in the study. Eligible patients were randomly assigned in a 3:1 ratio to the training and internal validation cohorts using computer-generated randomized numbers. The flowchart of the study is shown in **Figure 1A**. This study was approved by the Institutional Review Board of Henan Cancer Hospital (approval number: 2025-KY-0007-001), and an exemption of informed consent was obtained from the Institutional Review Board of Henan Cancer Hospital.

### Follow-up and outcomes

Patients with TNBC were included from the date of diagnosis to the date of death, or May 15, 2025, whichever came first. The endpoint of this study was OS. OS was defined as the number of days from the date of diagnosis to the date of death due to any cause or last follow-up.

### Statistical analysis

The chi-squared test or Fisher’s exact test was used to compare categorical variables. Quantitative variables are presented as medians with interquartile ranges (IQR), and statistical comparisons were made using Student’s T test or non-parametric Mann-Whitney U test. Kaplan-Meier analysis was used to estimate survival, and the log-rank test was used to compare differences between survival curves.

Variables with P < 0.05 in univariate Cox analysis were incorporated into multivariate Cox analysis to identify independent prognostic factors of TNBC in the training cohort. A nomogram for predicting OS was constructed based on the multivariable Cox results. The 1-year, 3-year, and 5-year receiver operating characteristic (ROC) curves were drawn to evaluate the predictive performance of the nomogram in the training and internal validation cohorts. A larger area under the ROC curve (AUROC) indicated greater predictive accuracy of the nomogram. Time-dependent ROC curves, corresponding AUC values, and Harrell’s C-indexes were utilized to measure and compare the performance of the nomogram, AJCC TNM stage, Miller-Payne (MP) system, and Ki-67 between the training and internal validation cohorts. Decision curve analysis (DCA) was performed to identify whether the nomogram could be deemed useful for clinical decision-making by comparing the net benefits at any threshold probability (13). All patients were stratified into three risk groups (low-risk, moderate-risk, and high-risk groups) according to two optimal cut-offs identified by the X-tile in the training cohort (14).

Statistical tests were two-sided, and statistical significance was set at P < 0.05. All statistical analyses were performed using SPSS version 26 and R version 4.5.0.

## Results

### Characteristics of the training cohort and internal validation cohort

A total of 804 TNBC patients who received NAT were selected. Among them, 603 (75%) patients were included in the training cohort and 201 (25%) patients were included in the internal validation cohort (**Figure 1A**). The baseline characteristics and therapeutic methods of the two cohorts are presented in **Table 1** and **Supplementary Table S1**. Notably, compared with the internal validation cohort, a higher proportion of patients in the training cohort had negative Her-2 immunohistochemical expression (P=0.04) and received adjuvant immunotherapy (P=0.03). The other characteristics were comparable between the two cohorts.

**Table 1:** Baseline clinicopathologic features in 804 patients.

Baseline characteristics	Enter cohort (n = 804)	Training cohort (n = 603)	Internal validation cohort (n = 201)	P value
Age (n = 804), median (IQR), y	49 (41-55)	49 (42-56)	49 (41-55)	0.94
Menopausal status (n = 804)				0.23
Premenopausal	451 (56.1%)	331 (54.9%)	120 (59.7%)	
Postmenopausal	353 (43.9%)	272 (45.1%)	81 (40.3%)	
Primary site (n = 804)				0.60
Right	365 (45.4%)	277 (45.9%)	88 (43.8%)	
Left	439 (54.6%)	326 (54.1%)	113 (56.2%)	
Pretreatment tumor size (n = 804), median (IQR), cm	3.0 (2.5-4.0)	3.0 (2.5-3.9)	3.0 (2.4-4.5)	0.07
Pretreatment lymph node metastasis (n = 804)				0.71
No	337 (41.9%)	255 (42.3%)	82 (40.8%)	
Yes	467 (58.1%)	348 (57.7%)	119 (59.2%)	
Clinical TNM staging (n = 804)				0.96
IA	35 (4.4%)	26 (4.3%)	9 (4.5%)	
IIA	308 (38.3%)	231 (38.3%)	77 (38.3%)	
IIB	255 (31.7%)	196 (32.5%)	59 (29.4%)	
IIIA	114 (14.2%)	83 (13.8%)	31 (15.4%)	
IIIB	4 (0.5%)	3 (0.5%)	1 (0.5%)	
IIIC	88 (10.9%)	64 (10.6%)	24 (11.9%)	
Her-2 immunohistochemical expression (n = 804)				**0.04**
0	313 (38.9%)	249 (41.3%)	64 (31.8%)	
1+	267 (33.2%)	197 (32.7%)	70 (34.8%)	
2+	224 (27.9%)	157 (26.0%)	67 (33.3%)	
Ki-67 (n = 793), median (IQR), %	70 (60-80)	70 (60-80)	70 (60-80)	0.09
Neoadjuvant immunotherapy (n = 804)				0.23
No	687 (85.4%)	510 (84.6%)	177 (88.1%)	
Yes	117 (14.6%)	93 (15.4%)	24 (11.9%)	
Surgery type (n = 804)				0.22
Modified radical mastectomy	513 (63.8%)	392 (65.0%)	121 (60.2%)	
Breast-conserving surgery	291 (36.2%)	211 (35.0%)	80 (39.8%)	
Pathological status (n = 804)				
non-pCR	412 (51.2%)	299 (49.6%)	113 (56.2%)	0.10
pCR	392 (48.8%)	304 (50.4%)	88 (43.8%)	
Skin invasion (n = 804)				0.80
No	799 (99.4%)	599 (99.3%)	200 (99.5%)	
Yes	5 (0.6%)	4 (0.7%)	1 (0.5%)	
Lymphovascular invasion (n = 804)				0.07
No	698 (86.8%)	531 (88.1%)	167 (83.1%)	
Yes	106 (13.2%)	72 (11.9%)	34 (16.9%)	
Perineural involvement (n = 804)				0.27
No	797 (99.1%)	599 (99.3%)	198 (98.5%)	
Yes	7 (0.9%)	4 (0.7%)	3 (1.5%)	
Post-mastectomy radiotherapy (n = 804)				0.15
No	143 (17.8%)	114 (18.9%)	29 (14.4%)	
Yes	661 (82.2%)	489 (81.1%)	172 (85.6%)	
Adjuvant immunotherapy (n = 804)				**0.03**
No	733 (91.2%)	542 (89.9%)	191 (95.0%)	
Yes	71 (8.8%)	61 (10.1%)	10 (5.0%)	
Follow-up duration (n = 804), median (IQR), m	28 (19-44)	28 (19-44)	29 (19-46)	0.70

P values ≤ 0.05 were considered significant and were marked in bold.

IQR, interquartile range.

The median follow-up period for the total population was 28 months (IQR, 19-44). The median follow-up periods for the training and internal validation cohorts were 28 months (IQR, 19-44) and 29 months (IQR, 19-46), respectively. In total, 41 (6.80%) patients died in the training cohort and 19 (9.45%) patients died in the internal validation cohort. No significant difference in survival was observed between the training and internal validation cohorts (P=0.234, **Figure 1B**).

### Univariate Cox analysis and multivariate Cox analysis

Univariate Cox proportional hazards regression analysis showed significantly longer survival in patients aged ≤ 65 years at diagnosis (P < 0.01, hazard ratio [HR] = 5.75, 95% CI=2.24-14.73), those with pretreatment tumor size ≤ 5cm (P=0.04, HR=2.22, 95% CI=1.06-4.64), and those without pretreatment lymph node metastasis (P < 0.01, HR=8.23, 95% CI=2.54-26.68). Furthermore, modified radical mastectomy (P=0.03, HR=0.41, 95%CI = 0.18-0.93), achieved pCR (P=0.01, HR=0.42, 95%CI = 0.21-0.81), no skin invasion (P < 0.01, HR=17.50, 95%CI = 5.36-57.12), no LVI (P < 0.01, HR=5.60, 95%CI = 3.00-10.45), and perineural involvement (P < 0.01, HR=13.30, 95%CI = 4.09-43.26) significantly improved prognosis. However, there was no significant association between OS and menopausal status (P=0.68, HR=1.14, 95%CI = 0.62-2.10), primary site (P=0.62, HR=1.17, 95%CI = 0.63-2.18), Her-2 immunohistochemical expression (compared to patients with Her-2 immunohistochemical expression 1+, P=0.65, HR=0.84, 95%CI = 0.40-1.77 or Her-2 immunohistochemical expression 2+, P=0.76, HR=0.89, 95%CI = 0.42-1.87), Ki-67 immunohistochemical expression (P=0.63, HR=1.00, 95%CI = 0.99-1.02), neoadjuvant immunotherapy (P=0.40, HR=0.60, 95%CI = 0.18-1.95), post-mastectomy radiotherapy (P=0.60, HR=1.25, 95%CI = 0.55-2.82), or adjuvant immunotherapy (P=0.46, HR=0.47, 95%CI = 0.06-3.45) (**Table 2**).

**Table 2:** Univariate and multivariate Cox analysis for predicting overall survival (OS) in 603 training cohort.

Risk characteristics	Univariable analysis			Multivariable analysis		
	HR	95%CI	P value	HR	95%CI	P value
Age						
≤ 65	Reference			Reference		
> 65	5.75	2.24-14.73	**≤ 0.01**	3.21	1.16-8.87	0.03
Menopausal status						
Premenopausal	Reference					
Postmenopausal	1.14	0.62-2.10	0.68			
Primary site						
Right	Reference					
Left	1.17	0.63-2.18	0.62			
Pretreatment tumor size, cm						
≤ 5	Reference			Reference		
> 5	2.22	1.06-4.64	**0.04**	2.31	1.03-5.15	**0.04**
Pretreatment lymph node metastasis						
No	Reference			Reference		
Yes	8.23	2.54-26.68	**≤ 0.01**	5.24	1.58-17.38	**0.01**
Her-2 immunohistochemical expression						
0	Reference		0.89			
1+	0.84	0.40-1.77	0.65			
2+	0.89	0.42-1.87	0.76			
Ki-67, % (+1%)	1.00	0.99-1.02	0.63			
Neoadjuvant immunotherapy						
No	Reference					
Yes	0.60	0.18-1.95	0.40			
Surgery type						
Breast-conserving surgery	Reference			Reference		
Modified radical mastectomy	0.41	0.18-0.93	**0.03**	0.80	0.33-1.93	0.62
Pathological status						
non-pCR	Reference			Reference		
pCR	0.42	0.21-0.81	**0.01**	0.82	0.37-1.80	0.61
Skin invasion						
No	Reference			Reference		
Yes	17.50	5.36-57.12	**≤ 0.01**	4.51	1.06-19.19	**0.04**
Lymphovascular invasion						
No	Reference			Reference		
Yes	5.60	3.00-10.45	**≤ 0.01**	2.59	1.15-5.84	**0.02**
Perineural involvement						
No	Reference			Reference		
Yes	13.30	4.09-43.26	**≤ 0.01**	2.94	0.67-12.92	0.15
Post-mastectomy radiotherapy						
No	Reference					
Yes	1.25	0.55-2.82	0.60			
Adjuvant immunotherapy						
No	Reference					
Yes	0.47	0.06-3.45	0.46			

P values ≤ 0.05 were considered significant and were marked in bold.

Multivariate Cox proportional hazards regression analysis demonstrated that age > 65 years (P=0.03, HR=3.21, 95%CI = 1.16-8.87), pretreatment tumor size > 5cm (P=0.04, HR=2.31, 95%CI = 1.03-5.15), pretreatment lymph node metastasis (P=0.01, HR=5.24, 95%CI = 1.58-17.38), skin invasion (P=0.04, HR=4.51, 95%CI = 1.06-19.19), and LVI (P=0.02, HR=2.59, 95%CI = 1.15-5.84) were independently significantly associated with decreased OS (**Table 2**).

### The prognostic nomogram for TNBC based on multivariable prognostic analysis

**Figure 2:**
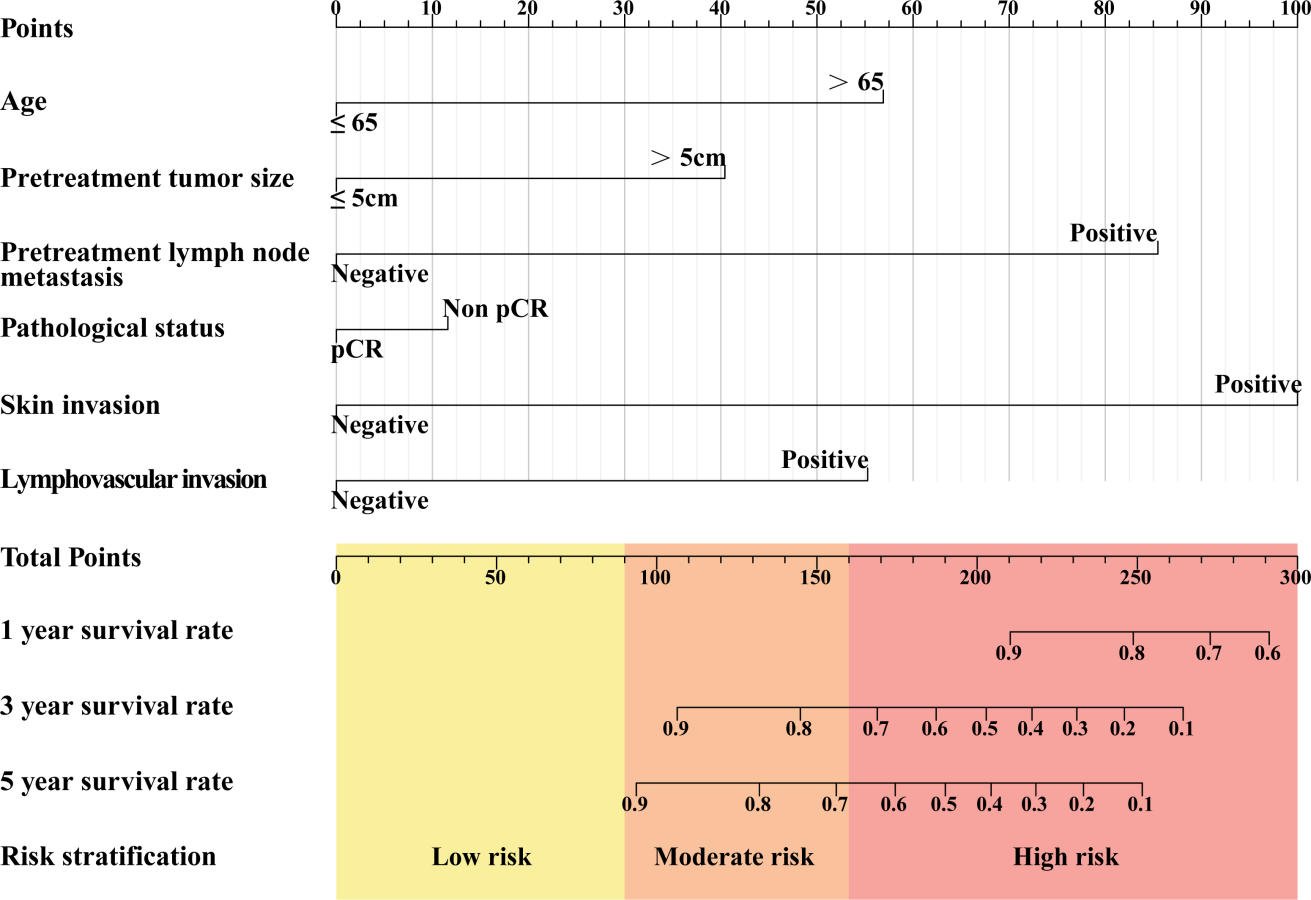
The prognostic nomogram model for predicting overall survival (OS) in triple-negative breast cancer (TNBC) patients treated with neoadjuvant therapy (NAT).

Age ≤ 65 years, pretreatment tumor size ≤ 5cm, no pretreatment lymph node metastasis, no skin invasion, and no LVI were identified as independent protective factors for patients with TNBC treated with NAT. In addition, pCR has been confirmed to be closely related to the independent prognosis of BC (7, 15, 16). Therefore, the pCR was incorporated into the prognostic nomogram. A prognostic nomogram for predicting OS was formulated by integrating all independent prognostic factors (**Figure 2**).

### Internal validation of the prognostic nomogram

**Figure 3:**
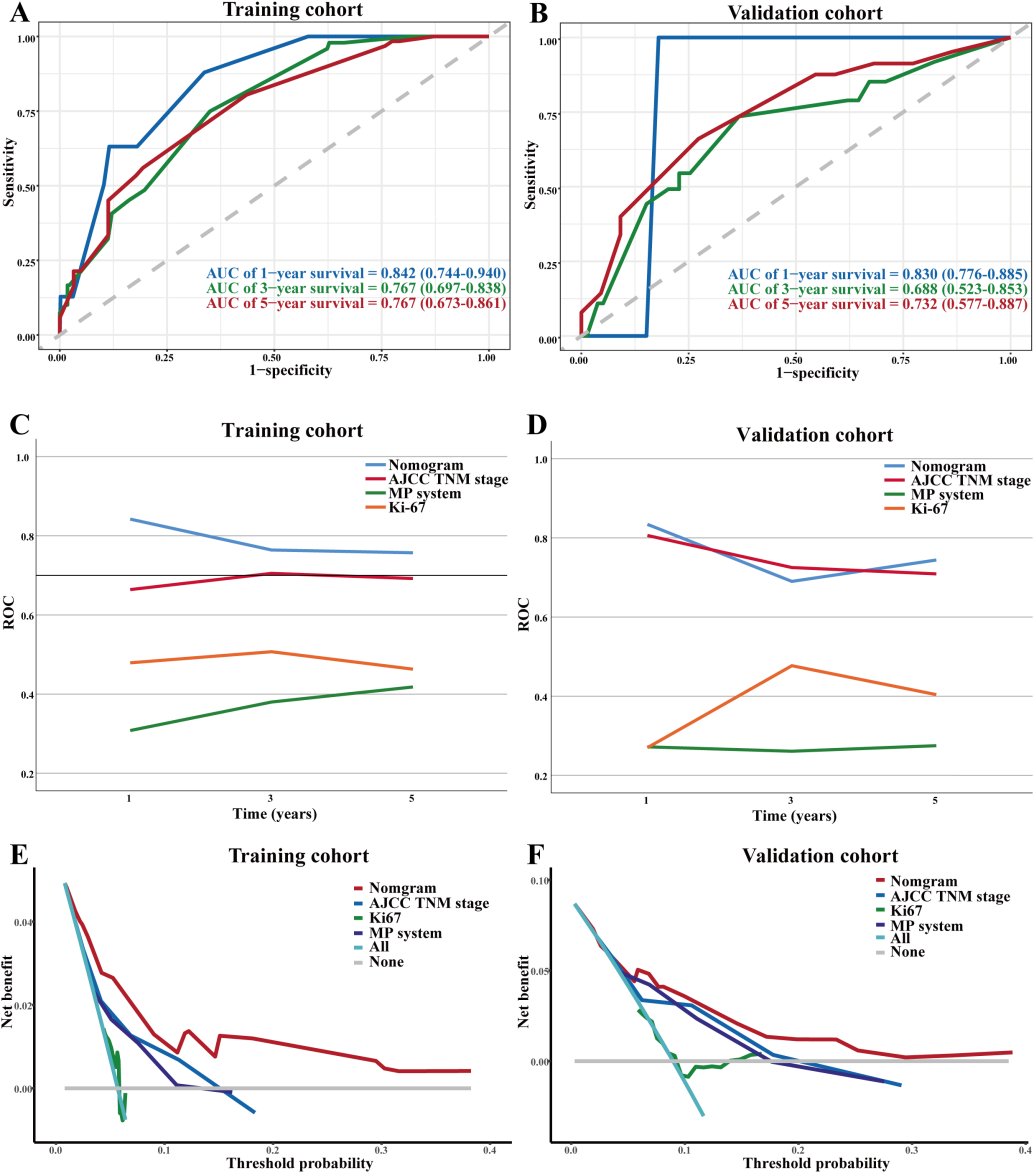
Evaluation of the specificity and sensitivity of the prognostic nomogram model for overall survival (OS) in triple-negative breast cancer (TNBC) patients receiving neoadjuvant therapy (NAT). receiver operating characteristic (ROC) analysis of the nomogram for predicting 1-year, 3-year, and 5-year OS in the training cohort **(A)** and internal validation cohort **(B)**. Time-dependent area under the ROC curve (AUC) values of the nomogram and other factors in the training cohort **(C)** and internal validation cohort **(D)**. decision curve analysis (DCA) analysis of the nomogram and other factors for predicting median OS in the training cohort **(E)** and internal validation cohort **(F)**.

The prognostic nomogram-predicted OS with 1-year, 3-year, and 5-year AUC was 0.842 (95% CI, 0.743-0.941), 0.764 (95% CI, 0.692-0.835) and 0.757 (95% CI, 0.662-0.852) respectively, in the training cohort, and 0.834 (95% CI, 0.781-0.888), 0.690 (95% CI, 0.532-0.848) and 0.744 (95% CI, 0.596-0.892) respectively, in the internal validation cohort (**Figures 3A, B**).

### Comparison of the predictive performance of the prognostic nomogram and other factors

The performance and discrimination of the prognostic nomogram and other factors (including AJCC TNM stage, MP system, and Ki-67 index) were compared (**Table 3**). The 1-year, 3-year, and 5-year AUC values and C-indexes of the prognostic nomogram were higher than those of the training and internal validation cohorts, indicating favorable performance and discrimination (**Figures 3C, D**, **Table 3**), indicating favorable performance and discrimination. DCA indicated that benefits of the prognostic nomogram robustly outperformed the other factors in the training and internal validation cohorts (**Figures 3E, F**).

**Table 3:** Comparison of the performance and discrimination of the current nomogram and other predictors in training and internal validation cohorts.

Cohort	Models	1-year AUC (95%CI)	3-year AUC (95%CI)	5-year AUC (95%CI)	C-index (95%CI)
Training cohort	Nomogram	0.842 (0.744-0.940)	0.767 (0.697-0.838)	0.767 (0.673-0.861)	0.780 (0.645-0.915)
	AJCC	0.666 (0.509-0.823)	0.708 (0.618-0.797)	0.698 (0.590-0.806)	0.705 (0.544-0.866)
	MP system	0.309 (0.125-0.493)	0.381 (0.282-0.480)	0.425 (0.312-0.538)	0.662 (0.478-0.846)
	Ki-67	0.478 (0.308-0.647)	0.507 (0.409-0.605)	0.463 (0.336-0.591)	0.513 (0.363-0.663)
Internal validation cohort	Nomogram	0.830 (0.776-0.885)	0.688 (0.523-0.853)	0.732 (0.577-0.887)	0.715 (0.475-0.955)
	AJCC	0.810 (0.759-0.862)	0.712 (0.552-0.871)	0.697 (0.523-0.870)	0.719 (0.472-0.966)
	MP system	0.279 (0.220-0.337)	0.257 (0.121-0.394)	0.284 (0.089-0.479)	0.706 (0.487-0.925)
	Ki-67	0.270 (0.210-0.331)	0.477 (0.323-0.632)	0.404 (0.210-0.599)	0.580 (0.321-0.839)

### Risk stratification of the prognostic nomogram

**Figure 4:**
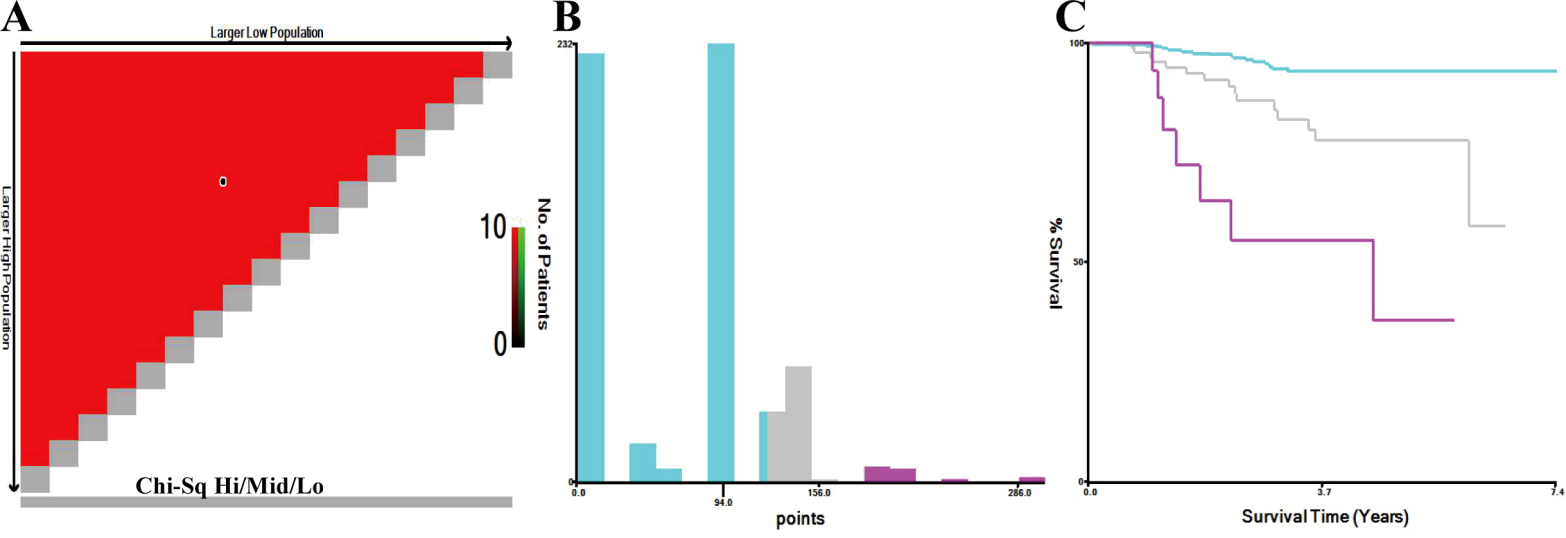
Identification of the optimal cut-offs for triple-negative breast cancer (TNBC) patients treated with neoadjuvant therapy (NAT) using X-tile software **(A–C)** in the training cohort.

**Figure 5:**
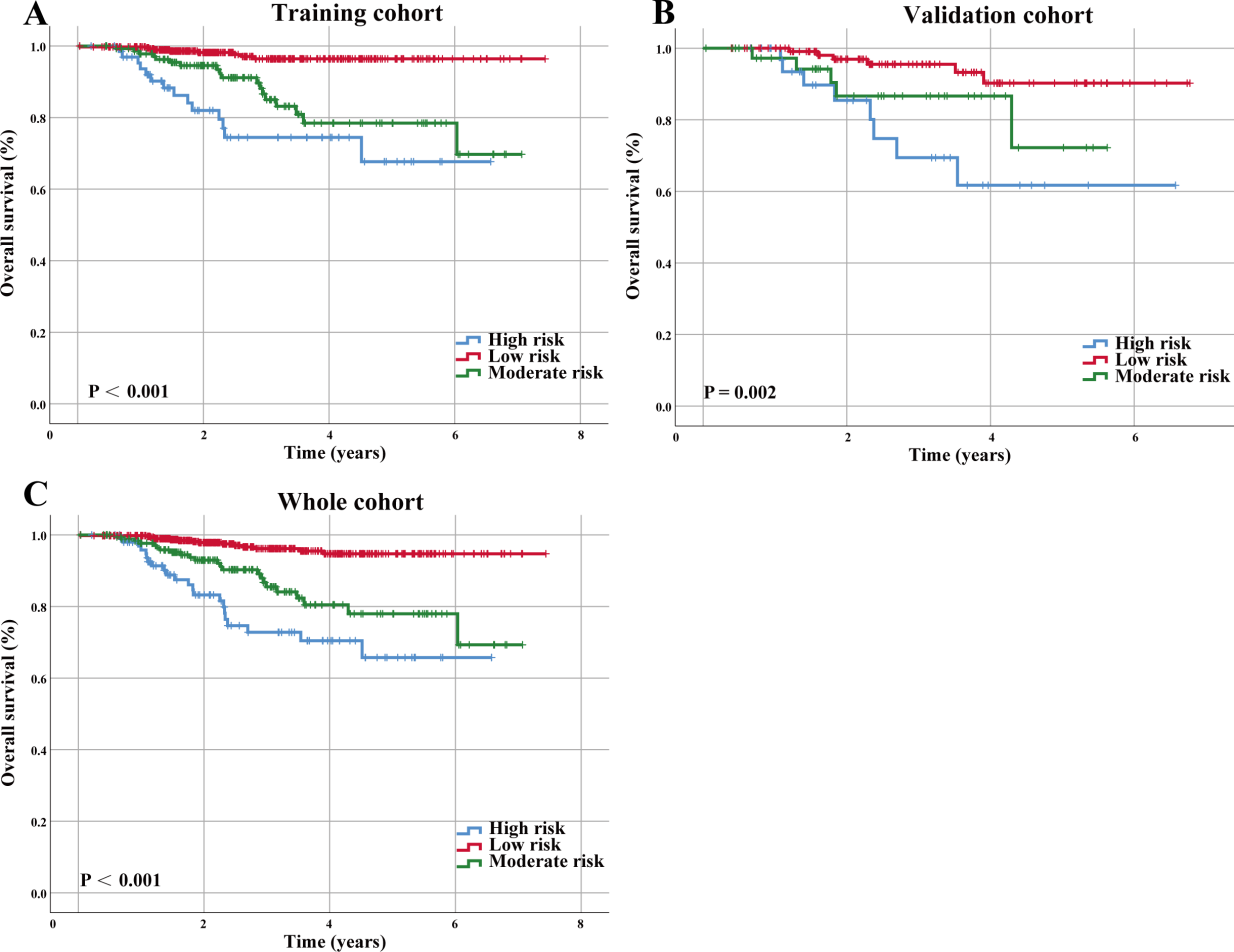
Kaplan-Meier survival analysis of three risk groups in the training cohort **(A)**, internal validation cohort **(B)** and whole cohort **(C)**.

We used X-tile software to generate two optimal cut-offs (90 and 160, **Figures 4A–C**), which divided TNBC into three groups with a highly significantly different probability of predicting OS (**Figure 2**): low-risk (total points ≤ 90, n = 393 in the training cohort, and n = 123 in the internal validation cohort), moderate-risk (90 < total points ≤160, n =143 in the training cohort, and n = 42 in the internal validation cohort), and high-risk (total points > 160, n = 67 in the training cohort, and n = 36 in the internal validation cohort). The Kaplan-Meier survival curves showed significant differences among the three risk groups in the training (P < 0.001), internal validation (P=0.002), and whole cohorts (P < 0.001) (**Figures 5A–C**).

### Performance of the risk stratification model in immunotherapy

The discriminative performance of the risk stratification model in immunotherapy was evaluated (**Table 4**). Compared with low-risk and moderate-risk groups, neoadjuvant immunotherapy (P=0.012) and adjuvant immunotherapy (P=0.015) were significantly fewer frequent in high-risk group. .

**Table 4:** Analysis of immunotherapy in low-risk, moderate-risk, and high-risk groups.

Risk characteristics	Low-risk group	Moderate-risk group	High-risk group	P value
	(n = 516)	(n = 185)	(n = 103)	
Neoadjuvant immunotherapy				**0.012**
No	427 (82.8%)	165 (89.2%)	95 (92.2%)	
Yes	89 (17.2%)	20 (10.8%)	8 (7.8%)	
Adjuvant immunotherapy				**0.015**
No	460 (89.1%)	173 (93.5%)	100 (97.1%)	
Yes	56 (10.9%)	12 (6.5%)	3 (2.9%)	

P values ≤ 0.05 were considered significant and were marked in bold.

**Table 5:** Summary of the prognostic models of triple-negative breast cancer (TNBC) with neoadjuvant therapy (NAT) from literatures and our study.

Author/Year	Cases	Endpoint	Model name	Factors	C-index	AUC	1-year AUC	3-year AUC	5-year AUC
Our study/2026	804	OS	Prognostic nomogram model	Age, pretreatment tumor size, pretreatment lymph node metastasis, pathological status, skin invasion, and lymphovascular invasion	0.780	-	0.842	0.767	0.767
Ende/2026 (32)	204	Good responder	Multigene model	31 genes	–	0.697	–	–	–
Cheng/2026 (33)	103	DFS, OS	DLRN model	Radiomics features	0.887, 0.800	-	-	-	-
Büyüktoka/2026 (34)	43	pCR	–	Magnetic resonance imaging-based features	–	0.75	–	–	–
Zheng/2026 (35)	103	DFS	-	Pathological type, axillary lymph node involvement, and peripheral halo	-	-	-	0.786	0.739
Chen/2025 (36)	124	pCR	Fusion radiomics model	Radiomics features	–	0.873	–	–	–
Liu/2025 (37)	90	pCR	Baseline and early treatment MRI model	Tumor unifocality, and early tumor shrinkage	-	0.88	-	-	-
Adrada/2025 (38)	264	pCR	Imaging- and tumor biomarker-based multivariable model	MRI-based TVR, Ki-67, and sTILs	–	0.84	–	–	–
Groheux/2025 (39)	57	pCR	FDG-PET/CT and multimodal machine learning model	PET, histopathological, genomic, and clinical features	-	0.82	-	-	-
Wang/2025 (40)	431	pCR	New immune-inflammatory-nutritional score	cT stage, Ki-67, and IIN score	–	0.827	–	–	–
Ma/2024 (41)	504	Rapid relapse	-	Age, pN stage, sTIL expression, and NAC response	-	0.938	-	-	-
Chung/2022 (42)	88	pCR	Integrative clinical model	NLR, PLR, echogenic halo, and tumor height-to-width ratio	–	0.877	–	–	–
Marmé/2021 (43)	1795	DFS, OS	CPS + EG scoring system	Pre-treatment clinical stage, post-treatment pathological stage, oestrogen-receptor status and grade	0.713, 0.749	-	-	-	-
Telli/2016 (44)	70	pCR, RCB	Homologous recombination deficiency score	Loss of heterozygosity, telomeric allelic imbalance, and large-scale state transitions	–	–	–	–	–

-, not mention.

## Discussion

Survival outcomes among TNBC patients who received NAT varied greatly. Therefore, the accurate prediction of individualized estimation and risk stratification in clinical trial design and practice is important. Based on 804 TNBC patients who received NAT, we developed a risk stratification nomogram model that can estimate individualized prognosis. The cut-off points separated the three distinct risk groups and had good predictive accuracy with high time-dependent AUROC values. The superiority and innovative aspects are as follows: (1) establishing the large sample nomogram model with a large sample to stratify the prognostic risk for TNBC with NAT; (2) validating the excellent performance of the risk stratification model in the training and internal validation cohorts; and (3) demonstrating the utility of the nomogram model for predicting OS in early-stage TNBC treated with NAT.

Age, pretreatment tumor size, pretreatment lymph node metastasis, pathological status, skin invasion, and LVI were independent predictors in patients with TNBC who received NAT (17–21). Pretreatment tumor size and pretreatment lymph node metastasis, which indicate primary and metastatic lymph node burden, respectively, have been identified as predictors in numerous studies (12, 22). Similarly, skin invasion and LVI have been consistently reported as predictors in numerous studies (23–26). Furthermore, several studies have consistently confirmed that pCR is an important prognostic indicator that outperforms other predictors (27, 28). In the present study, age > 65 years was associated with worse survival, which is inconsistent with the poor prognosis of young TNBC patients reported in the literature (29). This discrepancy may reflect the reduced tolerance to intensive NAT among older patients. Robust predictors were included in the present study, which partly contributed to the favorable performance of the model.

The optimal target population for NAT has been explored and determined in several randomized clinical trials; however, verification regarding its prognostic difference is lacking. This gap may produce unrecognized confounding factors that might potentially affect the results of these studies; therefore, a risk-stratification model for discriminating and diminishing heterogeneity is urgently needed (30, 31). Our nomogram model can separate TNBC patients who received NAT into different risk groups and can help clinicians select a potential high-risk cohort and conduct clinical trials to decrease the incidence of relapse. Currently, the AJCC TNM stage, which was originally introduced to predict survival in the context of therapy, is most widely used to guide the prognosis and treatment of TNBC (12). We compared our nomogram model with the AJCC TNM staging system and found that our model had better prediction accuracy. This finding does not imply that our model could replace the AJCC TNM staging system. Survival remains the most crucial outcome for patients with TNBC, and the AJCC TNM staging system has been specifically developed to predict survival. A more appropriate use of our model is to stratify patients who undergo NAT, making risk assessment more precise. Importantly, once identified as a “high-risk” patient, more attention should be paid to therapeutic strategies and disease monitoring.

In TNBC, a multitude of prognostic prediction models have been established; however, few models use OS as the primary endpoint (**Table 5**) (32–44). The C-index of our prognostic nomogram (C-index = 0.780) was comparable to that of the previously reported DLRN model (C-index = 0.800) (33) and CPS + EG scoring system (C-index = 0.749) (43). The prominent strengths of our study include the large-scale sample cohort (n = 804) and the integration of critical pathological factors, including pathological status, skin invasion, and LVI.

Immunotherapy, specifically anti-program cell death protein-1 (PD-1)/programmed death-ligand 1 (PD-L1), has emerged as program a transformative approach in the treatment of TNBC, offering new hope for patients previously faced with limited treatment options (45–47). Although immunotherapy was not included in the prognostic nomogram, chi-squared test analysis revealed that the high-risk group exhibited less neoadjuvant immunotherapy and adjuvant immunotherapy, suggesting that patients receiving immunotherapy may confer superior survival outcomes, consistent with current literature (2, 46, 48). Considering that only 14.6% and 8.8% of patients receive neoadjuvant immunotherapy and adjuvant immunotherapy (**Table 1**), further validation with a large sample size is needed.

Our study had several limitations. First, this study was a single-center retrospective study, which was limited by selection bias and lack of external validation; however, this shortcoming has been diminished by identifying the population using strict criteria. Second, needle biopsy for metastatic axillary nodes before NAT and/or evaluation of status in resected specimens was not routinely performed. Third, several studies have identified circulating tumor DNA, genomic information, and tumor-infiltrating lymphocytes in residual disease after NAT as independent prognostic predictors (49–53), but these factors were not evaluated in our study. Given these drawbacks, the application of the prognostic nomogram model needs to be further verified.

## Conclusions

The risk stratification nomogram model established in this study is the large sample risk stratification nomogram for predicting individual TNBC prognosis. With excellent performance and discriminative ability, the nomogram model could divide patients treated with NAT into three groups with significantly different survival rates. Therefore, the risk stratification nomogram might be useful for estimating the potential high-risk population of TNBC patients treated with NAT and identifying comparable candidates in clinical trials. Further verification is required in randomized controlled trials.

## Data Availability

The raw data supporting the findings of this study are available on request from the corresponding author, HG.

## Glossary

A: anthracycline
AUROC: area under the ROC curve
BC: breast cancer
C: cyclophosphamide
Cox: cox proportional-hazards model
DCA: decision curve analysis
ER: estrogen receptor
HER-2: human epidermal growth factor receptor 2
IQR: interquartile range
G: gemcitabine
LVI: lymphovascular invasion
MP: Miller-Payne
NAT: neoadjuvant therapy
N: vinorelbine
OS: overall survival
P: platinum
pCR: pathological complete response
PR: progesterone receptor
ROC: receiver operating characteristic
T: taxane
TNBC: triple-negative breast cancer
TNM: tumor-node-metastasis
X: xeloda.
